# Observer-Independent Assessment of Content Overlap in Mental Health Questionnaires: Large Language Model–Based Study

**DOI:** 10.2196/79868

**Published:** 2025-12-11

**Authors:** Annkathrin Böke, Hannah Hacker, Millennia Chakraborty, Luise Baumeister-Lingens, Jasper Vöckel, Julian Koenig, David HV Vogel, Theresa Katharina Lichtenstein, Kai Vogeley, Lana Kambeitz-Ilankovic, Joseph Kambeitz

**Affiliations:** 1 Department of Psychiatry and Psychotherapy, Faculty of Medicine and University Hospital of Cologne, University of Cologne, Kerpener Str. 62, Cologne, 50931, Germany, 49 22147887150; 2 Department of Child and Adolescent Psychiatry, Psychosomatics and Psychotherapy, Faculty of Medicine and University Hospital Cologne, University of Cologne, Cologne, Germany; 3 Department of Psychiatry and Psychotherapy, University Hospital Bonn, Bonn, Germany; 4 Cognitive Neuroscience (INM-3), Institute of Neuroscience and Medicine, Forschungszentrum Jülich, Jülich, Germany

**Keywords:** large language models, sentence Bidirectional Encoder Representations from Transformers, sBERT, GPT, questionnaires, scales, symptom overlap, content overlap

## Abstract

**Background:**

Mental disorders are frequently evaluated using questionnaires, which have been developed over the past decades for the assessment of different conditions. Despite the rigorous validation of these tools, high levels of content divergence have been reported for questionnaires measuring the same construct of psychopathology. Previous studies that examined the content overlap required manual symptom labeling, which is observer-dependent and time-consuming.

**Objective:**

In this study, we used large language models (LLMs) to analyze content overlap of mental health questionnaires in an observer-independent way and compare our results with clinical expertise.

**Methods:**

We analyzed questionnaires from a range of mental health conditions, including adult depression (n=7), childhood depression (n=15), clinical high risk for psychosis (CHR-P; n=11), mania (n=7), obsessive-compulsive disorder (n=7), and sleep disorder (n=12). Two different LLM-based approaches were tested. First, we used sentence Bidirectional Encoder Representations from Transformers (sBERT) to derive numerical representations (embeddings) for each questionnaire item, which were then clustered using k-means to group semantically similar symptoms. Second, questionnaire items were prompted to a Generative Pretrained Transformer to identify underlying symptom clusters. Clustering results were compared to a manual categorization by experts using the adjusted rand index. Further, we assessed the content overlap within each diagnostic domain based on LLM-derived clusters.

**Results:**

We observed varying degrees of similarity between expert-based and LLM-based clustering across diagnostic domains. Overall, agreement between experts was higher than between experts and LLMs. Among the 2 LLM approaches, GPT showed greater alignment with expert ratings than sBERT, ranging from weak to strong similarity depending on the diagnostic domain. Using GPT-based clustering of questionnaire items to assess the content overlap within each diagnostic domain revealed a weak (CHR-P: 0.344) to moderate (adult depression: 0.574; childhood depression: 0.433; mania: 0.419; obsessive-compulsive disorder [OCD]: 0.450; sleep disorder: 0.445) content overlap of questionnaires. Compared to the studies that manually investigated content overlap among these scales, the results of this study exhibited variations, though these were not substantial.

**Conclusions:**

These findings demonstrate the feasibility of using LLMs to objectively assess content overlap in diagnostic questionnaires. Notably, the GPT-based approach showed particular promise in aligning with expert-derived symptom structures.

## Introduction

Mental health questionnaires are essential tools to assess psychological and psychiatric conditions, offering insights into symptom presence, severity, frequency, and duration [1]. Over the last decades, a wide range of questionnaires has been developed, each requiring rigorous validation of its validity and reliability [2]. Particularly, the analysis of a questionnaire’s content is crucial in order to assure that it validly measures the intended construct [3]. However, a surprising degree of content divergence has been reported among questionnaires designed to measure the same construct [1,4-11]. For example, a comparison of 4 depression questionnaires revealed that while items assessing general, somatic, and positive symptoms were consistently included, 4 factors (anxiety, positive emotions, interpersonal functioning, and performance impairment) were unique to individual questionnaires [4]. A more detailed analysis of the content overlap of 7 depression questionnaires revealed only a weak similarity among the questionnaires, with 40% of symptoms appearing in just one questionnaire [6]. Similar results have been reported for questionnaires of childhood depression, clinical high risk for psychosis (CHR-P), mania, obsessive-compulsive disorder (OCD), and sleep disorder [7-11]. Thus, it is questionable if questionnaires designed to measure the same construct can be used interchangeably [12]. This lack of interchangeability has important implications, as it may compromise comparability across studies, affect the reproducibility of findings, and introduce bias in clinical practice and research.

These more detailed analyses of questionnaire similarity quantified the content overlap by determining whether items of different questionnaires assess the same symptoms, following a method proposed by Fried [6]. This is done through a manual categorization of questionnaire items, where researchers assign each questionnaire item (eg, “Trouble concentrating on things, such as reading the newspaper or watching television”) to a symptom category (eg, “cognitive deficits”). This process is both time-consuming and observer-dependent, leading to inconsistencies and limiting the scalability and reliability of content overlap assessments. Therefore, an objective and more automated process is needed to make the evaluation of content overlap more accessible.

With advances in artificial intelligence, large language models (LLMs) have emerged as powerful tools for analyzing and generating text [13-15]. A key feature of LLMs is their ability to transform text into numerical representations within a high-dimensional vector space, so-called embeddings [14]. Sentences with similar content typically have embeddings that are in close proximity to each other within this vector space. For example, “I have trouble concentrating” is expected to be located closer to “I find it hard to focus” than to “I feel sad.” Thus, LLMs offer an effective method for capturing the underlying semantic structure and quantifying the semantic similarities of sentences [16]. Combined with clustering, an unsupervised machine learning technique, embeddings can be used to group texts with semantically similar content [13,17,18]. In addition to embedding generation, LLMs can also be guided through prompting, that is, eliciting meaningful text outputs by providing specific instructions or questions to the LLM [19]. Although static embeddings generated without prompting are consistent, they often lack interpretability; in contrast, the more flexible approach of prompting produces auto-generated, interpretable results but can be inconsistent and depends heavily on the quality of the prompt [20-23]. LLMs’ capability of text analysis has been of interest in psychological research, for example, to validate constructs of psychological questionnaires, to predict the relationship between questionnaire items, and to generate new questionnaires [24-29]. Given their ability to quantify semantic similarities, LLMs present a promising alternative for automating and standardizing the assessment of questionnaire content overlap.

Thus, in this study, we used LLMs to objectively quantify the content overlap of mental health questionnaires that have previously been analyzed through manual categorization. Therefore, we first evaluated whether LLMs can group questionnaire items in a manner comparable to clinical experts by using two approaches. First, we used a state-of-the-art LLM [14] to derive static embeddings of questionnaire items and used unsupervised machine learning to group items assessing similar symptoms. Second, prompting was used to determine symptoms underlying questionnaire items. In a second step, we assessed the content overlap across mental health questionnaires based on the LLM-derived groupings. Our goal is to demonstrate that LLMs can effectively cluster questionnaire items based on symptoms, thereby improving our knowledge about mental health questionnaires with a focus on their heterogeneity.

## Methods

### Questionnaires

This analysis was based on a selection of mental health questionnaires that have been previously investigated with respect to their content overlap [6-11]. This included questionnaires for adult depression (n=7) [30-36], childhood depression (n=15) [30,37-50], CHR-P (n=11) [51-61], mania (n=7) [62-68], OCD (n=7) [69-75], and sleep disorder (n=12) [76-86]. The questionnaires were included based on their frequency in literature, inclusion in reviews, and citation count. The full details about the selection process can be found in the previous publications. A summary of all questionnaires can be found in Table 1. It has to be noted that the Depression and Anxiety in Youth Scale (DAYS) [87], the Multiscore Depression Inventory for Children (MDI-C) [88], the Reynolds Adolescent Depression Scale (RADS) [89], and the Eppendorf Schizophrenia Inventory (ESI) [90] were neither publicly available nor purchasable. Hence, these questionnaires could not be included in the analysis. Further, we did not include the Child Behavior Checklist and Youth Self Report [91] as these are not specifically designed to assess the risk for psychosis.

**Table 1.** Summary of questionnaires.

Questionnaire	Reference	Rating type	Items
Adult depression			
Beck Depression Inventory (BDI-II)	[30]	SR^a^	21
Hamilton Rating Scale for Depression (HDRS)	[31]	OR^b^	17
Center of Epidemiological Scales Depression Scale (CES-D)	[32]	SR	20
Inventory of Depressive Symptoms (IDS)	[33]	SR, OR	60
Quick Inventory of Depressive Symptoms (QIDS)	[34]	SR	32
Montgomery-Åsberg Depression Rating Scale (MADRS)	[35]	OR	10
Zung Self-Rating Depression Scale (SDS)	[36]	SR	20
Childhood depression			
BDI-II	[30]	SR	21
Depression Self Rating Scale (DSRS)	[37]	SR	18
Center for Epidemiological Studies Depression Scale for Children (CESD-C)	[38]	SR	20
Children’s Depression Scale (CDS)	[39]	SR	66
Children’s Depression Inventory (CDI)	[40]	SR	12
The Mood and Feelings Questionnaire (MFQ)	[41]	SR	33
Weinberg Screening Affective Scale Long Form (WSAS)	[42]	SR	56
Reynolds Child Depression Scale (RCDS)	[43]	SR	30
Depression Anxiety Stress Scales (DASS) depression subscale	[44]	SR	14
Revised Child Anxiety and Depression Scale (RCADS)	[45]	SR	47
Patient Health Questionnaire (PHQ)	[46]	SR	10
Kutcher Adolescent Depression Scale (KADS)	[47]	SR	16
The Adolescent Depression Rating Scale (ADRS)	[48]	OR	10
Neuro-QOL–Pediatric Depression	[49]	SR	8
PROMIS Pediatric Depressive Symptoms	[50]	SR	14
CHR-P^c^			
Behavior Assessment System for Children Atypicality Scale (BASC Atypicality)	[51]	SR	10
Brief Self-Report Questionnaire for Screening Putative Pre-Psychotic States (BSQSP)	[52]	SR	15
Community Assessment of Psychic Experiences (CAPE-42)	[53]	SR	42
Early Detection Primary Care Checklist (EDPCCL)	[54]	OR	20
Early Recognition Inventory based on IRAOS (ERIraos)	[55]	SR	15
Perceptual and cognitive aberrations scale (PCA)	[56]	SR	9
Prodromal Questionnaire (PQ-16)	[57]	SR	16
PROD-screen	[58]	SR, OR	21
PRIME Screen—Revised (PS-R)	[59]	SR	12
Self-screen Prodrome	[60]	SR	32
Youth Psychosis At-Risk Questionnaire – Brief (YPARQ-B)	[61]	SR	28
Mania			
Young Mania Rating Scale (YMRS)	[62]	OR	11
Mood Disorder Questionnaire (MDQ)	[63]	SR	13
Clinician‐ Administered Rating Scale for Mania (CARS‐M)	[64]	OR	15
Bech‐Rafaelsen Mania Rating Scale (BRMRS)	[65]	OR	11
Hypomanic Checklist 32–(HCL‐32)	[66]	SR	32
Bipolar Spectrum Disorder Scale (BSDS)	[67]	SR	19
Mood Swings Questionnaire (MSQ)	[68]	SR	27
OCD^d^			
Children’s Florida Obsessive Compulsive Inventory (C-FOCI)	[69]	SR	22
Children’s Obsessional Compulsive Inventory-Revised-Self Report (ChOCI-R-S)	[70]	SR	34
Children’s Yale-Brown Obsessive Compulsive Scale (CY-BOCS)	[71]	OR	85
Leyton Obsessional Inventory Child Version (LOI-CV)	[72]	SR	44
Obsessive Compulsive Inventory Child Version (OCI-CV)	[73]	SR	21
OCD Family Functioning Scale (OFF)	[74]	SR	42
Short Obsessive–Compulsive Disorder Screener in children and adolescents (SOCS)	[75]	SR	7
Sleep disorder			
Auckland Sleep Questionnaire (ASQ)	[76]	SR	34
Basic Nordic Sleep Questionnaire (BNSQ)	[77]	SR	21
Global Sleep Assessment Questionnaire (GSAQ)	[78]	SR	11
Holland Sleep Disorders Questionnaire (HSDQ)	[79]	SR	32
Iowa Sleep Disturbances Inventory (ISDI)	[80]	SR	86
Oviedo Sleep Questionnaire (OSQ)	[81]	SR	10
Pittsburgh Sleep Quality Index (PSQI)	[82]	SR	10
Sleep Disorder Questionnaire (SDQ)	[82]	SR	175
Sleep Disorders Symptom Checklist 17 (SDS-CL-17)	[83]	SR	17
Sleep Disorders Symptom Checklist 25 (SDS-CL-25)	[84]	SR	25
Sleep Disorders Symptom Checklist 50 (SDS-CL-50)	[85]	SR	50
Sleep Symptom Checklist (SSC)	[86]	SR	21

a SR: self-rating.

b OR: observer-rating.

c CHR-P: clinical high risk for psychosis.

d OCD: obsessive-compulsive disorder.

**Figure 1.**
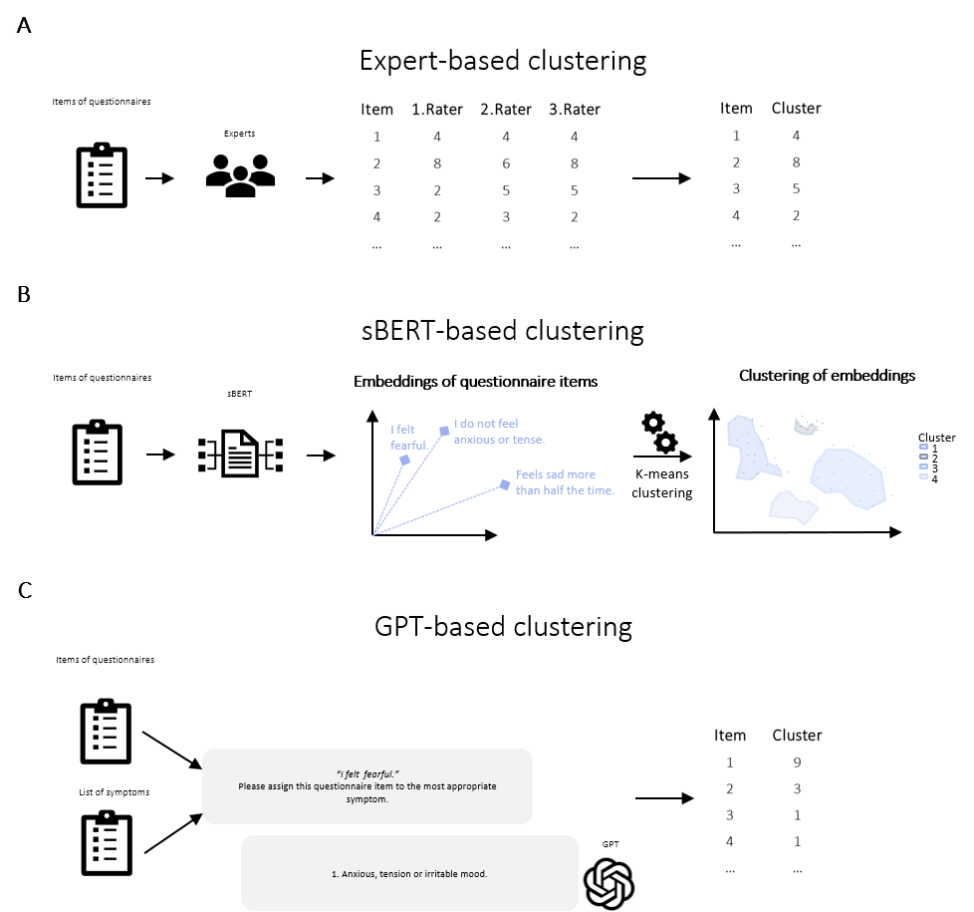
Clustering of mental health questionnaire items. (A) Expert-based clustering. (B) Sentence-Bidirectional Encoder Representations from Transformers–based clustering. (C) Generative Pretrained Transformer–based clustering. GPT: Generative Pretrained Transformer; sBERT: Sentence-Bidirectional Encoder Representations from Transformers.

For each diagnostic domain (adult depression, childhood depression, CHR-P, mania, OCD, and sleep disorder) we identified the respective core symptoms based on the *Diagnostic and Statistical Manual of Mental Disorders, 5th edition (DSM-5*) [92]. For CHR-P symptoms, we extracted the core symptoms from the Structured Interview for Psychosis-Risk Syndromes (SIPS) [8]. Thereby, symptom features were listed as individual symptoms (eg, from the *DSM-5* item “Feelings of worthlessness or excessive or inappropriate guilt,” the symptoms “Feelings of worthlessness” and “Feelings of guilt” were extracted). Each questionnaire item was assigned to one of the core symptoms by 3 of 10 clinical experts independently (Figure 1A). All experts are working in the field of mental health as clinicians, psychotherapists, and researchers. Questionnaires were assigned to experts according to their field of expertise. Each element of a questionnaire to which a participant or observer must respond was considered as an item. Thereby, subitems were consolidated into one item. Items that were not related to symptoms but to assessment quality (eg, “These answers represent my honest feelings”) were removed from the analysis. If an item assessed multiple core symptoms, symptoms were combined and categorized with a more general symptom (eg, “There is no change from my usual appetite” can refer to both “decreased appetite” and “increased appetite,” which were combined as “changes in appetite”). In case an item could not be assigned to a core symptom from the *DSM-5* or SIPS, an additional symptom was added to the list of core symptoms (eg, “I feel very bored” refers to “boredom”).

### Similarity of Expert- and Embedding-Based Item Grouping

For each diagnostic domain, 3 clinical experts were independently provided with a list of questionnaire items and were instructed to assign each item to one of the core symptoms. Questionnaire items were assigned to core symptoms based on the highest agreement among expert ratings. In case no clear consensus was reached, the raters discussed and agreed upon the most appropriate categorization. Based on this, all items of questionnaires of the same diagnostic domain were clustered.

To cluster items by underlying symptoms using LLMs, we used 2 approaches. First, sentence embeddings of questionnaire items were derived from the pretrained sentence Bidirectional Encoder Representations from Transformers (sBERT) model “all-mpnet-base-v2” [14] (Figure 1B). The open-access sBERT model, trained on 160 gigabytes of English text corpora, was selected based on its superior performance in sentence encodings across 14 benchmark tasks and its recognition as one of the best validated models [93]. The resulting sentence embeddings of a fixed dimensionality of 768 were clustered using k-means clustering. The number of clusters was set to the same number as in the expert-based analysis. Second, OpenAI’s third-generation Generative Pretrained Transformer (GPT) model “GPT-3.5-Turbo-0125” [94] was used to assign each questionnaire item to a corresponding symptom (Figure 1C). Its wide availability, computational efficiency, and high performance at comparatively low cost made it a suitable choice for this study [95]. Each item was presented to the model alongside the list of core symptoms, and the model was prompted to assign the item to the most appropriate symptom. Based on the model’s responses, items of each diagnostic domain were grouped according to their assigned symptom.

Similarity of clustering within each diagnostic domain, among the 3 experts and between the experts’ highest-agreement cluster (hereafter referred to as expert-based clustering) and the two LLM-based clustering approaches, was quantified using the Adjusted Rand Index (ARI), a widely used metric for evaluating agreement between clustering solutions [96,97]. The ARI ranges from -0.5 to 1.0, where negative values indicate clustering discordance lower than chance, values near zero suggest random clustering, and values approaching one reflect near-perfect agreement. In the absence of established guidelines for interpreting the strength of the ARI, we applied the classification used for correlation coefficients: very weak (0.00‐0.19), weak (0.20‐0.39), moderate (0.40‐0.59), strong (0.60‐0.79), and very strong (0.80‐1.00). First, we evaluated the similarity in ratings for each pair of raters and computed the mean similarity within each diagnostic domain. Second, the similarity between expert-based and both the sBERT- and GPT-based clustering solutions was evaluated across the diagnostic domains using the ARI. Given that both self- and observer-rated questionnaires were included in the analysis and the rating types of questionnaires exert a strong influence on how items are phrased, we additionally examined the similarity between the expert-based clusterings and both LLM-based clusterings separately for rating types. As a control of the sBERT-based approach, sentence embedding vectors were randomly permuted, and the analysis was repeated (1000 repetitions) as described before.

### Content Overlap of Questionnaires

In a second step, the content overlap of questionnaires within each diagnostic domain was calculated based on the method introduced by Fried [6] but using the GPT-based clustering approach. Specifically, for each diagnostic domain, the Jaccard index was calculated for each pair of questionnaires to determine their overlap of content. The Jaccard index is calculated by dividing the number of shared items between 2 clusters by the total number of items present in both clusters. The resulting values range from 0, indicating no overlap, to 1, representing complete overlap. Similar to Fried [6], we defined the strength of the Jaccard index as follows: very weak (0.00‐0.19), weak (0.20‐0.39), moderate (0.40‐0.59), strong (0.60‐0.79), and very strong (0.80‐1.0).

### Ethical Considerations

This study did not involve human participants, medical records, patient information of any kind, or secondary data analyses. Thus, the study did not meet the criteria for a review by an institutional review board, and no ethical approval was required.

## Results

### Questionnaires

A total of 23 symptoms of adult depression, 30 symptoms of childhood depression, 40 symptoms of CHR, 29 symptoms of mania, 27 symptoms of OCD, and 45 symptoms of sleep disorder were identified in the questionnaires based on core criteria from the *DSM-5* or SIPS (Table S1 inMultimedia Appendix 1). A more detailed graphical representation of the number of items assigned to each cluster by the 3 clustering approaches can be found in Figures S1-6 in Multimedia Appendix 1. It should be noted that defining the expert-based clustering solution based on the highest agreement among experts and limiting the analysis to self-rating (SR) or observer-rating (OR) questionnaires reduced the number of identified symptoms in some of the diagnostic domains (Table 2).

**Table 2.** Number of questionnaires, items, and identified symptoms for diagnostic domain and rating type.

Diagnostic domain	Questionnaires	Items	Symptoms
All			
Adult depression	7	180	23
Childhood depression	15	370	27
CHR-P^a^	11	220	38
Mania	8	131	29
OCD^b^	7	256	26
Sleep disorder	12	493	41
SR^c^			
Adult depression	5	107	21
Childhood depression	14	360	27
CHR-P	10	179	37
Mania	5	94	25
OCD	6	170	21
Sleep disorder	12	493	41
OR^d^			
Adult depression	2	73	20
Childhood depression	1	9	9
CHR-P	1	20	15
Mania	3	37	17
OCD	1	85	25

a CHR-P: clinical high risk for psychosis.

b OCD: obsessive-compulsive disorder.

c SR: self-rating.

d OR: observer-rating.

### Similarity of Expert- and Embedding-Based Item Grouping

**Figure 2.**
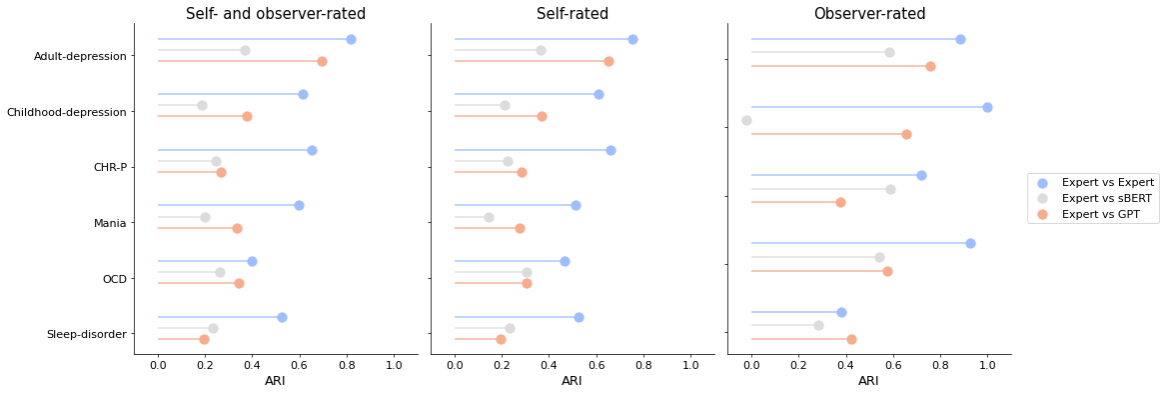
Adjusted Rand Index comparing clustering solutions of questionnaire items between expert raters, between expert-based to sBERT-based clustering solutions, and between expert-based to GPT-based clustering solutions. CHR-P: clinical high risk for psychosis; GPT: Generative Pretrained Transformer; OCD: obsessive-compulsive disorder; sBERT: Sentence-Bidirectional Encoder Representations from Transformers.

Between the 3 raters, we observed a very strong similarity for adult depression questionnaires (mean ARI: 0.819), a strong similarity for childhood depression questionnaires (mean ARI: 0.616), and CHR-P questionnaires (mean ARI: 0.654), and a moderate similarity for mania questionnaires (mean ARI: 0.597), OCD questionnaires (mean ARI: 0.401), and sleep disorders questionnaires (mean ARI: 0.527) (Table 3, Figure 2). Similarities between individual raters can be found in Figures S1-7 in Multimedia Appendix 1.

**Table 3.** Adjusted Rand Index comparing clustering solutions of questionnaire items between expert raters, between expert-based to sBERT-based clustering solutions, and between expert-based to GPT-based clustering solutions.

Diagnostic domain	Expert to expert	sBERT^a^ to expert		GPT^b^ to expert
	mean ARI	ARI	95% CI	ARI
All				
Adult depression	0.819	0.371	–0.001 to 0.000	0.694
Childhood depression	0.616	0.188	0.000 to 0.001	0.379
CHR-P^c^	0.654	0.245	0.000 to 0.001	0.266
Mania	0.597	0.213	–0.001 to 0.000	0.334
OCD^d^	0.401	0.265	0.000 to 0.001	0.345
Sleep disorder	0.527	0.235	–0.000 to 0.000	0.195
SR^e^				
Adult depression	0.752	0.366	–0.001 to 0.001	0.651
Childhood depression	0.610	0.213	0.000 to 0.001	0.367
CHR-P	0.661	0.227	–0.001 to 0.001	0.286
Mania	0.511	0.143	–0.001 to 0.001	0.275
OCD	0.468	0.304	–0.002 to –0.001	0.306
Sleep disorder	0.527	0.235	–0.000 to 0.000	0.195
OR^f^				
Adult depression	0.884	0.583	–0.003 to –0.001	0.759
Childhood depression	1.000	−0.023	–0.009 to 0.010	0.656
CHR-P	0.718	0.589	–0.004 to 0.005	0.378
Mania	0.925	0.540	–0.004 to 0.001	0.573
OCD	0.379	0.286	0.001 to 0.003	0.423

a sBERT: Sentence-Bidirectional Encoder Representations from Transformers.

b GPT: Generative Pretrained Transformer.

c CHR-P: clinical high risk for psychosis.

d OCD: obsessive-compulsive disorder.

e SR: self-rating.

f OR: observer-rating.

Similarity between expert-based and sBERT-based clusterings varied across the diagnostic domains. The ARI ranged from 0.188 (for childhood depression questionnaires) to 0.371 (for adult depression questionnaires), indicating a very weak to weak similarity (Table 3). Including solely SR items, the mean ARI ranged between a very weak (0.143 for mania questionnaires) and weak similarity (0.366 for adult depression). For OR questionnaires, we observed a similarity lower than chance (ARI: −0.023) for childhood depression questionnaires. Across the other domains, we observed ARIs in the range of a weak (0.286 for OCD questionnaires) to moderate similarity (0.589 for CHR-P questionnaires). Irrespective of diagnostic domains and rating types, the mean ARI exceeded the 95% CI indicating agreement above chance level, with the exception of the childhood depression OR questionnaires.

Additionally, we observed a varying similarity between expert-based and GPT-based clustering solutions. We observed a very weak (0.195 for sleep disorder questionnaires) to strong (0.694 for adult depression questionnaires) similarity across the different diagnostic domains (Table 3). When focusing on SR items, the ARI varied from 0.195 (for sleep disorder questionnaires) to 0.651 (for adult depression questionnaires), indicating again a very weak to strong similarity. Including solely OR items, we observed a weak (0.378 for CHR-P questionnaires) to strong (for adult depression questionnaires) similarity.

### Content Overlap of Questionnaires

**Figure 3.**
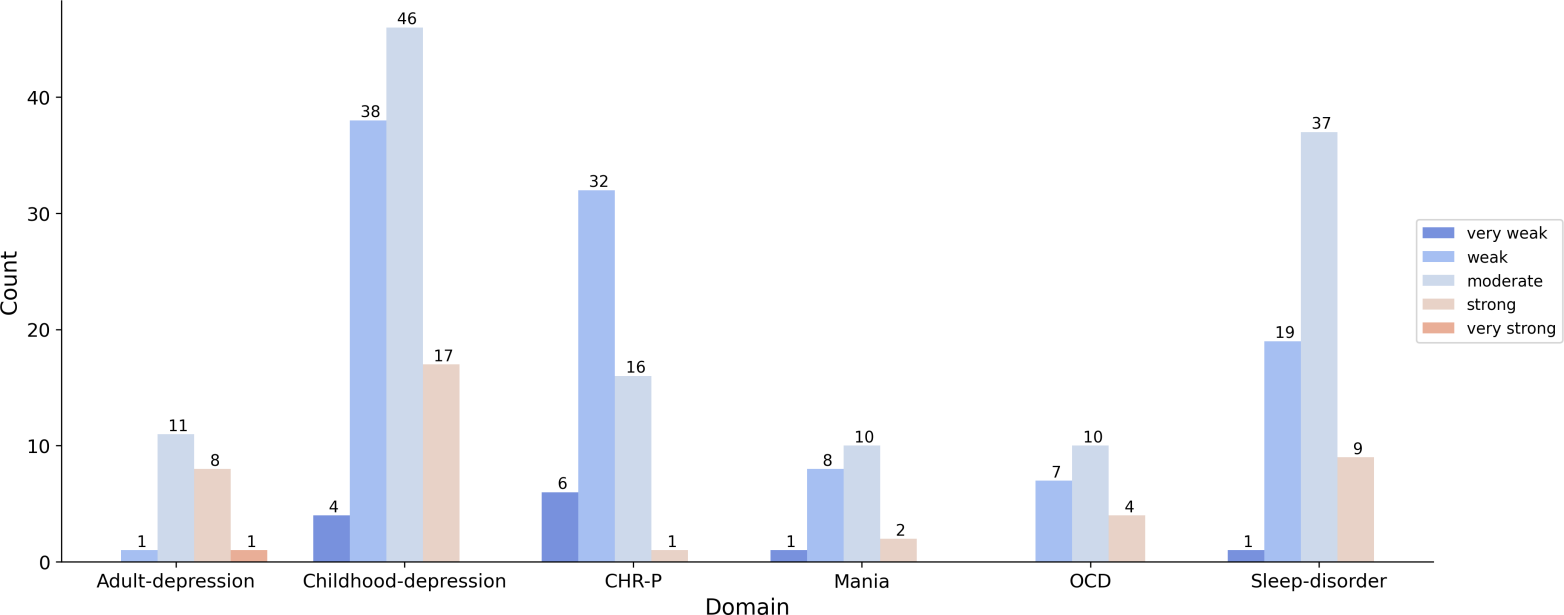
Content overlap of questionnaires within each diagnostic domain. CHR-P: clinical high risk for psychosis; OCD: obsessive-compulsive disorder.

Using the method introduced by Fried [6] but adapted to the GPT-based clustering approach to assess the content overlap of questionnaires, we observed a weak content overlap for CHR-P questionnaires (mean Jaccard index: 0.344) and a moderate content overlap for adult depression questionnaires (mean Jaccard index: 0.574), childhood depression questionnaires (mean Jaccard index: 0.443), mania questionnaires (mean Jaccard index: 0.419), OCD questionnaires (mean Jaccard index: 0.457), and sleep disorder questionnaires (mean Jaccard index: 0.461). An overview of the observed content overlap within each diagnostic domain is presented in Figure 3. Interactive sunburst plots showing a more detailed overview of the content overlap of the questionnaires in each diagnostic domain can be found on GitHub [98].

As similarity between the expert-based and GPT-based clustering was highest for adult depression questionnaires, we focused on the content overlap of these questionnaires in greater detail. The number of symptoms and the average Jaccard index per questionnaire can be found in Table 4.

**Table 4.** Average overlap of clusters across adult depression questionnaires.

Questionnaire	Number of items	Number of symptoms	Mean Jaccard index
BDI-II^a^	21	12	0.549
CES-D^b^	20	15	0.601
HDRS^c^	17	11	0.445
IDS^d^	60	14	0.623
MADRS^e^	10	8	0.567
QIDS^f^	32	12	0.653
SDS^g^	20	13	0.579

a BDI-II: Beck Depression Inventory.

b CES-D: Center of Epidemiological Studies Depression Scale.

c HDRS: Hamilton Rating Scale for Depression.

d IDS: Inventory of Depressive Symptoms.

e MADRS: Montgomery-Åsberg Depression Rating Scale.

f QIDS: Quick Inventory of Depressive Symptoms.

g SDS: Zung Self-Rating Depression Scale.

**Figure 4.**
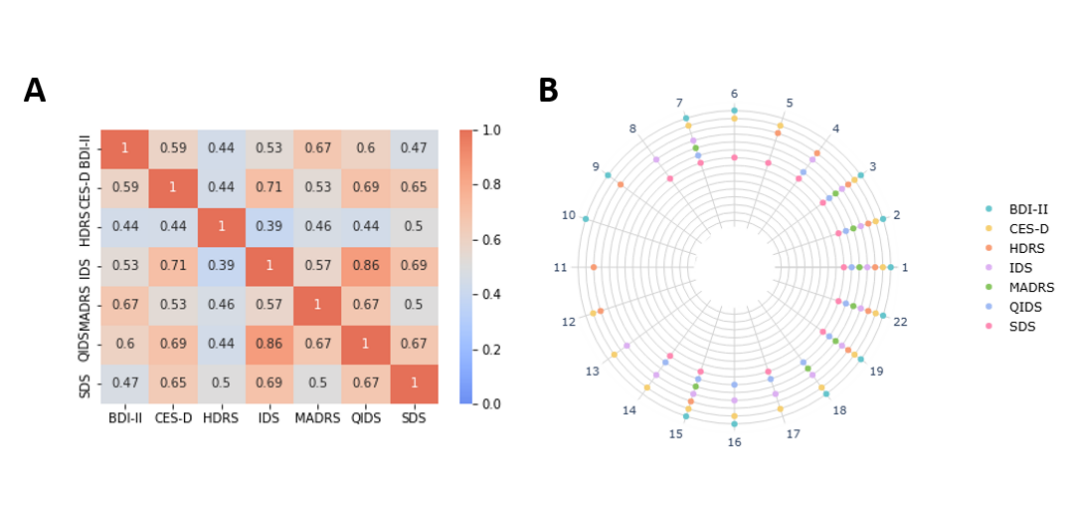
Content overlap of adult depression questionnaires. (A) Jaccard indices of each pair of adult depression questionnaires. (B) Occurrence of the 28 clusters across the depression questionnaires. The circle indicates that the questionnaire contained items belonging to this cluster. Colors of circles correspond to the questionnaire. BDI-II: Beck Depression Inventory; CES-D: Center of Epidemiological Studies Depression Scale; HDRS: Hamilton Rating Scale for Depression; IDS: Inventory of Depressive Symptoms; MADRS: Montgomery-Åsberg Depression Rating Scale; QIDS: Quick Inventory of Depressive Symptoms; SDS: Zung Self-Rating Depression Scale.

A total of 20 symptoms were found in questionnaire items of adult depression questionnaires using GPT-based clustering. Of these, 15/20 symptoms (75%) were found in the CES-D. Around 8/20 symptoms (40%) were identified in the Montgomery-Åsberg Depression Rating Scale (MADRS). Six symptoms were featured across all questionnaires (Figure 3). One symptom was exclusively represented in the BDI-II, while another item appeared only in the Hamilton Rating Scale for Depression (HDRS). Across all adult depression questionnaires, the mean content overlap was 0.574. For a detailed overview of the Jaccard indices, see Table 4 and Figure 4A. The content overlap of Inventory of Depressive Symptoms (IDS) and Quick Inventory of Depressive Symptoms (QIDS) was highest, whereas the lowest overlap was found between IDS and HDRS. Details of symptoms found in questionnaires can be found in Figure 4B.

## Discussion

### Principal Findings

To the best of our knowledge, this is the first study to use LLMs to assess the content overlap of mental health questionnaires. First, we aimed to compare expert-based clustering of questionnaire items to two observer-independent automatic approaches using LLMs: (1) clustering sentence embeddings generated by sBERT using k-means, and (2) prompting the questionnaire items directly to the GPT model. Across the different diagnostic domains, we found a moderate to very strong agreement between expert raters. Although similarity between expert-based and sBERT-based clustering was above chance, GPT-based clustering was in general more aligned with expert-based clustering except for sleep disorder questionnaires. In a second step, we calculated the content overlap of questionnaires within each diagnostic domain based on the GPT clustering. We observed weak (CHR-P questionnaires) to moderate (adult depression, childhood depression, mania, OCD, and sleep disorder questionnaires) content overlap.

### Similarity of Expert- and Embedding-Based Item Grouping

Generally, our findings demonstrate that LLMs such as sBERT and GPT can be effectively used to cluster questionnaire items based on content. Both models have previously been used to assess the validity of questionnaires and surveys [24,25,29]. Using a fine-tuned sBERT model to generate semantic embeddings of the International Personality Item Pool questionnaires, Wulff and Mata [29] demonstrated that these embeddings can predict a questionnaire’s empirical internal structure, convergent and divergent validity, and detect its structural fidelity. Similarly, the fine-tuned sBERT model SurveyBot3000 was able to infer correlations between questionnaire items and the intercorrelation of questionnaires of the American Psychological Association (APA) PsycTests corpus [24]. Further, Huang et al [25] used a GPT model to generate semantic embeddings of two gratitude questionnaires. Similarity between each questionnaire pair was then clustered to infer semantic similar items and identify redundancy within questionnaires. Our study extends these applications by highlighting LLMs’ utility in assessing content overlap in mental health questionnaires. Notably, we found that directly prompting items to GPT is particularly effective for generating meaningful content-based groupings of questionnaire items. Similarly, Petukhova [16] observed that GPT models outperform static embedding models like sBERT in clustering and semantic understanding tasks. Specifically, when combined with prompt engineering, GPT models offer advantages in generative reasoning over the fixed representations generated by sBERT [23]. Compared to methods that require generating and clustering embeddings from static models like sBERT, GPT prompting is not only more flexible but also easier to implement, as it does not require additional preprocessing or fine-tuning. Thus, GPT prompting appears to be a promising tool for rapidly obtaining an overview of the content overlap in mental health questionnaires. This application will likely further improve with future GPT models, as newer versions show increased ability to detect psychological constructs [99].

### Content Overlap of Questionnaires

Compared to previous studies, we observed a higher overlap between the questionnaires with the exception of sleep disorder questionnaires [6-11]. This is likely due to differences in the number of identified symptoms. Compared to previous studies, we identified fewer symptoms across all diagnostic domains, which may have resulted in a less fine-grained analysis and, consequently, an increased apparent content overlap. However, using more nuanced symptoms was not feasible, as some items lack specificity; for example, “I sleep a lot less than usual” can reflect both initial and middle insomnia. Nevertheless, we observed a substantial heterogeneity between questionnaires across all diagnostic domains, which strengthens previous observations of content divergence between questionnaires. Such heterogeneity of content is not inherently problematic. On the contrary, variability across questionnaires can be beneficial; it allows clinicians and researchers to capture complementary information, adapt to different contexts, and contribute to scientific advancement [100,101]. However, to fully leverage this variability, it is essential to understand the distinct strengths and applications of each questionnaire. This study demonstrates a method for facilitating the identification of content overlap, thereby making this process more accessible.

Focusing on the content overlap of adult depression questionnaires in greater detail revealed that the overlap of questionnaires is moderate (0.574), indicating that the results from one questionnaire may only partially generalize to others. In line with the observations of Fried [6], the content of the QIDS demonstrated the highest mean similarity with other questionnaires, but we observed the lowest mean similarity for the HDRS, not the CES-D. Related to this, we identified idiosyncratic items in the HDRS (and BDI-II) but not in the CES-D. Several studies have reported that the HDRS and BDI-II capture idiosyncratic symptoms [4,6,101]. In line with Fried [6], our GPT-based clustering identified only items related to feelings of punishment in the BDI-II, whereas items related to hypochondriasis appeared exclusively in the HDRS. However, additional idiosyncratic symptoms were identified in other questionnaires, and these differ from those reported by Shafer [4]. These differences may again be attributed to a less fine-grained symptom structure. Overall, our findings demonstrate that GPT-based content overlap analysis of questionnaires is not only feasible but also fast and computationally efficient, making it a promising approach for large-scale questionnaire comparisons. In the future, this approach will facilitate an extended investigation of questionnaire content overlap by incorporating additional instruments and enable broadening the analysis to questionnaires assessing other mental health disorders, such as anxiety disorders or posttraumatic stress disorder.

### Limitations

Several limitations have to be addressed. First, the number of items and symptoms was not consistent across the diagnostic domains. The discrepancy in the number of identified clusters may have influenced the results. However, reducing the number of clusters, and thus the number of identified symptoms, would decrease the specificity of symptoms and thus lower the accuracy of the content overlap analysis [6]. Further, the semantic representation of an LLM is influenced by its training data [25]. Both LLMs used in this study were trained on publicly available texts (eg, web pages, books, or Wikipedia) but not clinical datasets (eg, diagnostic manuals). This might have influenced the model’s representation of questionnaire items. However, both LLMs were shown to contain reliable general psychiatric knowledge and were able to classify mental health conditions without fine-tuning [102-104]. In future work, it would be valuable to compare the clustering performance of pretrained and fine-tuned models. Moreover, while the present study as well as several previous publications indicate that semantic embeddings can capture some aspects of medical terms and psychological concepts [26,102,105], it needs to be acknowledged that such LLM-based representations are most likely far from complete and can potentially be biased due to a number of reasons [106]. Thus, although LLMs can facilitate efficient and low-effort creation of questionnaire content, human expertise remains essential for reviewing and interpreting their outputs. Finally, only English-language questionnaires were included in this study. Whether LLMs can effectively cluster questionnaire items in other languages remains to be tested.

### Conclusion

In summary, our study demonstrates the feasibility of using LLMs to assess the content overlap in mental health questionnaires. In particular, prompting GPT models provides a novel and objective approach for evaluating similarity across questionnaires. It is important to note that human expertise remains essential for reviewing and interpreting the outputs of LLM. Although our findings differed somewhat from previous content overlap analyses, these differences were not substantial. Future content analysis could benefit from LLMs fine-tuned on a corpus of psychological text data. Nonetheless, this study demonstrates a novel application of LLMs in the field of mental health research.

## Supplementary material

10.2196/79868 Multimedia Appendix 1 Overview of identified symptoms in questionnaires and the number of items assigned to each cluster by the different approaches.

## Abbreviations

ARI: Adjusted Rand Index
BDI-II: Beck Depression Inventory
CES-D: Center of Epidemiological Studies Depression Scale
CHR-P: clinical high risk for psychosis
DAYS: Depression and Anxiety in Youth Scale
DSM-5: Diagnostic and Statistical Manual of Mental Disorders, 5th edition
ESI: Eppendorf Schizophrenia Inventory
GPT: Generative Pretrained Transformer
HDRS: Hamilton Rating Scale for Depression
IDS: Inventory of Depressive Symptoms
LLM: large language model
MADRS: Montgomery-Åsberg Depression Rating Scale
MDI-C: Multiscore Depression Inventory for Children
OCD: obsessive-compulsive disorder
OR: observer-rating
QIDS: Quick Inventory of Depressive Symptoms
RADS: Reynolds Adolescent Depression Scale
sBERT: sentence Bidirectional Encoder Representations from Transformers
SDS: Zung Self-Rating Depression Scale
SIPS: Structured Interview for Psychosis-Risk Syndromes
SR: self-rating
