# Supplementary material for: Observer-Independent Assessment of Content Overlap in Mental Health Questionnaires: Large Language Model–Based Study
Source: JMIR AI. 2025 Dec 11;4:e79868. doi: 10.2196/79868 (PMC12697914; doi:10.2196/79868)
Supplement: Multimedia Appendix 1 [file ai-v4-e79868-s001.docx]

This is a Multimedia Appendix to a full manuscript published in the JMIR mental health.

Table 1 shows a detailed overview of identified symptoms in questionnaires of diagnostic domains.

**Table S1.** List of symptoms identified in questionnaires. CHR-P: clinical high risk of psychosis. OCD: obsessive-compulsive disorder.

| Adult depression | |
| --- | --- |
| **1** | Anxious, tension or irritable mood |
| **2** | Changes in appetite |
| **3** | Changes in sleeping pattern |
| **4** | Changes in weight |
| **5** | Changes of speech or thought |
| **6** | Crying |
| **7** | Difficulty in concentration or decision-making |
| **8** | Diurnal variation |
| **9** | Feelings of guilt |
| **10** | Feelings of punishment |
| **11** | Hypochondriasis |
| **12** | Insight |
| **13** | Interpersonal rejection sensitivity |
| **14** | Loneliness |
| **15** | Loss of interest or pleasure |
| **16** | Low self-esteem or feelings of worthlessness |
| **17** | Mood reactivity |
| **18** | Pessimism |
| **19** | Psychomotor retardation or agitation |
| **20** | Tiredness or fatigue |
| **21** | Sadness or depressed mood |
| **22** | Somatic complaints |
| **23** | Anxious, tension, or irritable mood |
|  |  |
| Childhood depression | |
| **1** | Anxious, tension or irritable mood |
| **2** | Interpersonal rejection sensitivity |
| **3** | Hallucinations or delusion |
| **4** | Feelings of punishment |
| **5** | Boredom |
| **6** | Changes in appetite |
| **7** | Changes in weight |
| **8** | Compulsion |
| **9** | Difficulty in concentration or decision-making difficulty |
| **10** | Crying |
| **11** | Diminished interest or pleasure in activities |
| **12** | Feelings of guilt |
| **13** | Relationship to family |
| **14** | Lonliness |
| **15** | Social withdrawal |
| **16** | Sadness or depressed mood |
| **17** | Loss of interest or pleasure |
| **18** | Low self-esteem or feelings of worthlessness |
| **19** | Motivation |
| **20** | Psychomotor retardation or agitation |
| **21** | Pessimism |
| **22** | Anxious, tension, or irritable mood |
| **23** | Psychosocial functioning |
| **24** | Sleep disturbances |
| **25** | Tiredness or fatigue |
| **26** | Somatic complains |
| **27** | Somatic complaints |
| **28** | Stupor |
| **29** | Suicidal ideation |
| **30** | Worry |
|  |  |
| CHR-P | |
| **1** | Anxiety, panic, fears and phobias |
| **2** | Avolition |
| **3** | Bizarre thinking |
| **4** | Compulsive motor rituals |
| **5** | Diminished interest in pleasurable activities |
| **6** | Disorganized communication |
| **7** | Drug use |
| **8** | Dysphoric mood |
| **9** | Experience of emotions and self |
| **10** | Expression of emotions |
| **11** | Family history of mental illness |
| **12** | Feelings of irritability, hostility or rage |
| **13** | Feelings of restlessness, agitation or tension |
| **14** | Feelings of worthlessness or guilt |
| **15** | First-rank symptoms |
| **16** | General concern |
| **17** | Grandiose ideas |
| **18** | History of mental illness |
| **19** | Ideational richness |
| **20** | Impaired tolerance to normal stress |
| **21** | Impairment in personal hygiene |
| **22** | Impairment in social, work or school functioning |
| **23** | Impulsivity |
| **24** | Inappropriate affect |
| **25** | Lack of spontaneity and flow of conversation |
| **26** | Manic excitement |
| **27** | Non-persecutory ideas of reference |
| **28** | Obsessions |
| **29** | Odd behavior or appearance |
| **30** | Overvalued beliefs |
| **31** | Perceptual abnormalities or hallucinations |
| **32** | Perplexity or delusional mood |
| **33** | Preoccupied with or interactive with own thoughts |
| **34** | Sleep and appetite disturbances |
| **35** | Social anhedonia |
| **36** | Somatic ideas |
| **37** | Suicidal thoughts |
| **38** | Suspiciousness, paranoid thinking or persecutory idea of reference |
| **39** | Trouble with focus or attention |
| **40** | Unstable mood |
|  |  |
| Mania | |
| **1** | Appearance |
| **2** | Concentration problems |
| **3** | Decreased need for sleep |
| **4** | Depressive mood |
| **5** | Heightened senses |
| **6** | Increased energy |
| **7** | Disinhibition |
| **8** | Disorientation |
| **9** | Duration of symptoms |
| **10** | Elevated mood |
| **11** | Engaging in new projects |
| **12** | Family history |
| **13** | Grandiosity, delusions or hallucinations |
| **14** | Impaired social or occupational functioning |
| **15** | Increased goal-directed activity |
| **16** | Increased social activity |
| **17** | Inflated self-esteem |
| **18** | Irritable mood or aggressive behavior |
| **19** | Loss of energy |
| **20** | Loss of insight |
| **21** | More talkative |
| **22** | Poor judgment |
| **23** | Psychomotor agitation |
| **24** | Sexual interest |
| **25** | Shifts in mood or energy |
| **26** | Substance use |
| **27** | Suicidal ideation |
| **28** | Thought disorder |
| **29** | Weight gain |
|  | |
| OCD | |
| **1** | Fear of harm |
| **2** | Aggressive obsession or compulsion |
| **3** | Avoiding behaviors |
| **4** | Causes anxiety or distress |
| **5** | Functional impairment |
| **6** | Checking compulsion |
| **7** | Cleaning compulsion |
| **8** | Contamination obsession |
| **9** | Counting compulsion |
| **10** | Forbidden or taboo thoughts |
| **11** | Feelings of disgust |
| **12** | Perfectionism |
| **13** | Resistance |
| **14** | General urges |
| **15** | Hair-pulling |
| **16** | Hoarding objects |
| **17** | Intolerance of uncertainty |
| **18** | Things need to look, feel or sound "just right" |
| **19** | List making |
| **20** | Ordering compulsion |
| **21** | Symmetry obsession |
| **22** | Physical appearance obsession |
| **23** | Repetitive behavior |
| **24** | Rituals |
| **25** | Things need to look, feel or sound 'just right' |
| **26** | Time-consuming |
| **27** | Touching |
|  |  |
| Sleep disorder | |
| **1** | Alteration of circadian system |
| **2** | Alterations of desired sleep-wake rhythm |
| **3** | Altered oneiric activity |
| **4** | Amnesia |
| **5** | Autonomic nervous system dysfunction |
| **6** | health condition |
| **7** | Breath abnormalities |
| **8** | Cataplexy |
| **9** | Cognitive impairments |
| **10** | Functional impairment |
| **11** | Complex motor behaviors |
| **12** | General causes of sleep disturbances |
| **13** | Mood disturbances |
| **14** | Psychological condition |
| **15** | Daytime distress |
| **16** | Fatigue or daytime sleepiness |
| **17** | Daytime sleep |
| **18** | Disorientation |
| **19** | Family history of sleep disorders |
| **20** | Nonrestorative sleep |
| **21** | Working hours |
| **22** | General sleep disturbances |
| **23** | Maintaining insomnia |
| **24** | Health condition |
| **25** | Hypnopomia |
| **26** | Initial insomnia |
| **27** | Lapses into sleep |
| **28** | Late insomnia |
| **29** | Long sleep time |
| **30** | General sleep disturbances |
| **31** | Medical consultation |
| **32** | Nightmares |
| **33** | Overall sleep quality |
| **34** | Relaxation techniques |
| **35** | Restless legs |
| **36** | Sleep efficiency |
| **37** | Alterations of desired sleep-wake rhythm |
| **38** | Sleep inertia |
| **39** | Sleep terrors |
| **40** | Sleep type |
| **41** | Sleep walking |
| **42** | Snoring |
| **43** | Substance use |
| **44** | Teeth grinding |
| **45** | Vocalization |

A detailed graphical representation of the number of items assigned to each cluster by the expert-based and sBERT-based, and GPT-based clustering methods can be found in the Figure S1-6.


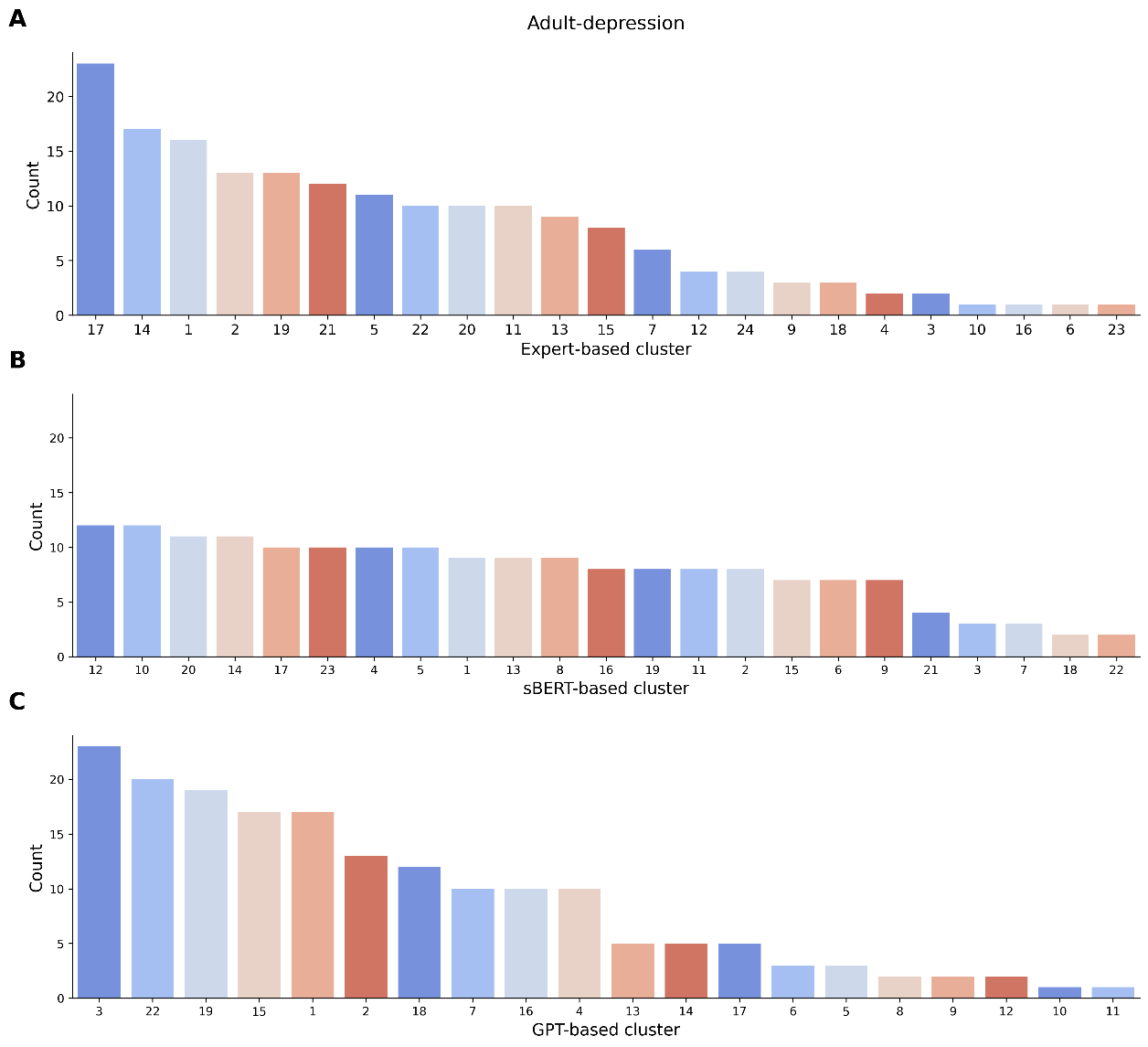


**Figure S1.** Number of items of adult depression questionnaires assigned to each cluster by (**A**) the expert-based and (**B**) the embedding-based clustering methods. sBERT: Sentence - Bidirectional Encoder Representations from Transformers. GPT: Generative Pre-Trained Transformer.


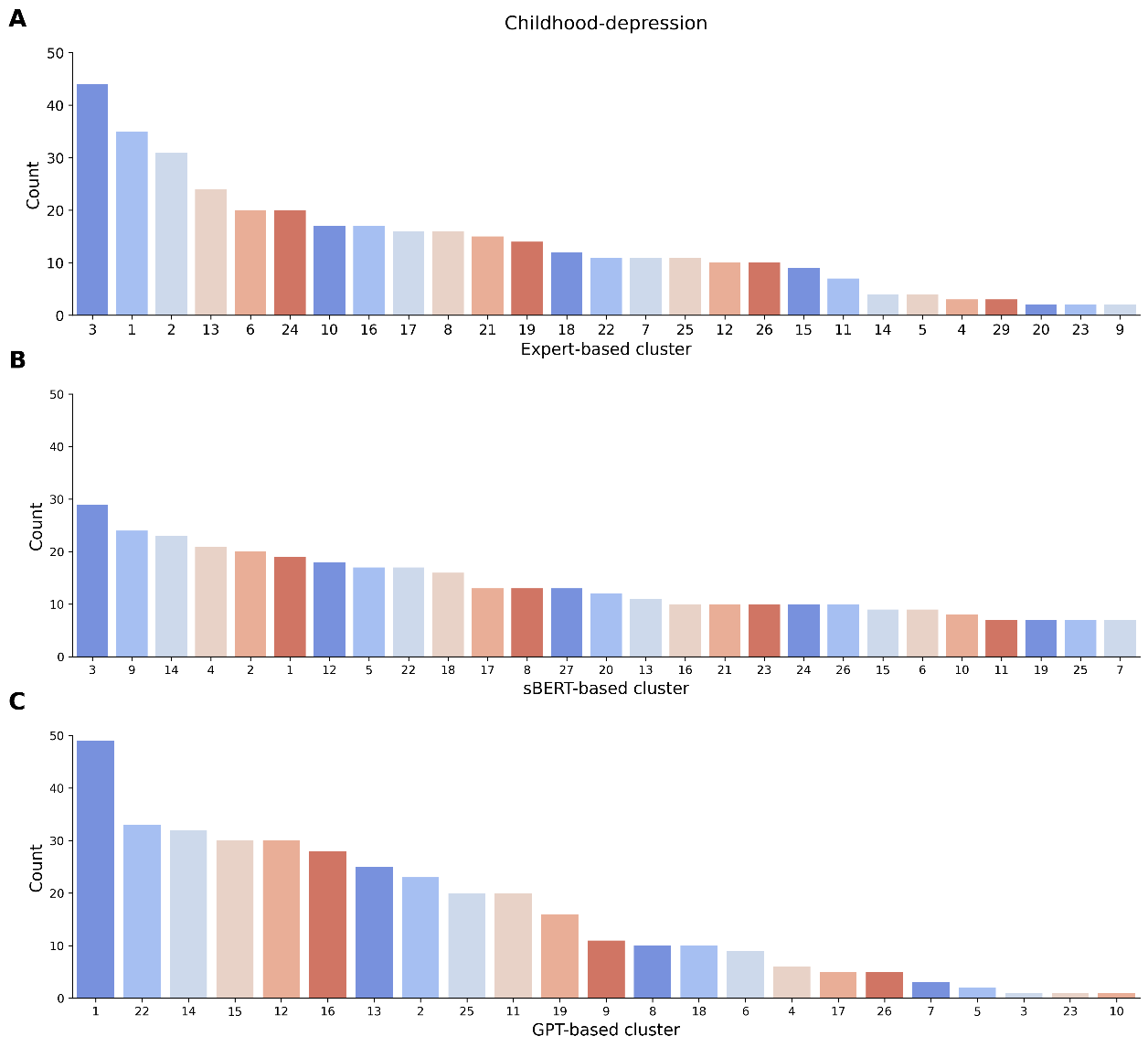


**Figure S2.** Number of items of childhood depression questionnaires assigned to each cluster by (**A**) the expert-based and (**B**) the embedding-based clustering methods. sBERT: Sentence - Bidirectional Encoder Representations from Transformers. GPT: Generative Pre-Trained Transformer.


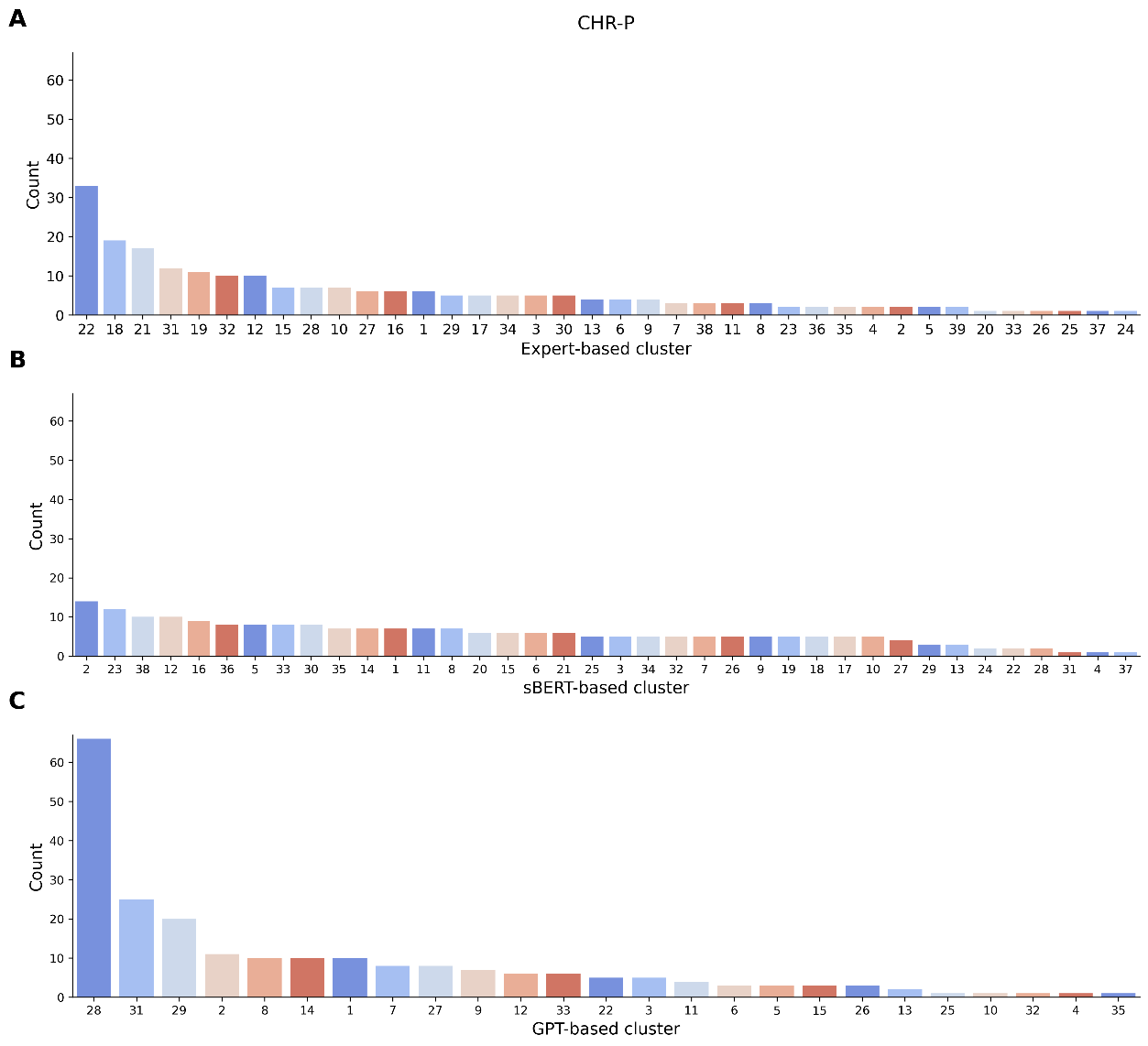


**Figure S3.** Number of items of clinical high risk for psychosis (CHR-P) questionnaires assigned to each cluster by (**A**) the expert-based and (**B**) the embedding-based clustering methods. BERT: Sentence - Bidirectional Encoder Representations from Transformers. GPT: Generative Pre-Trained Transformer.


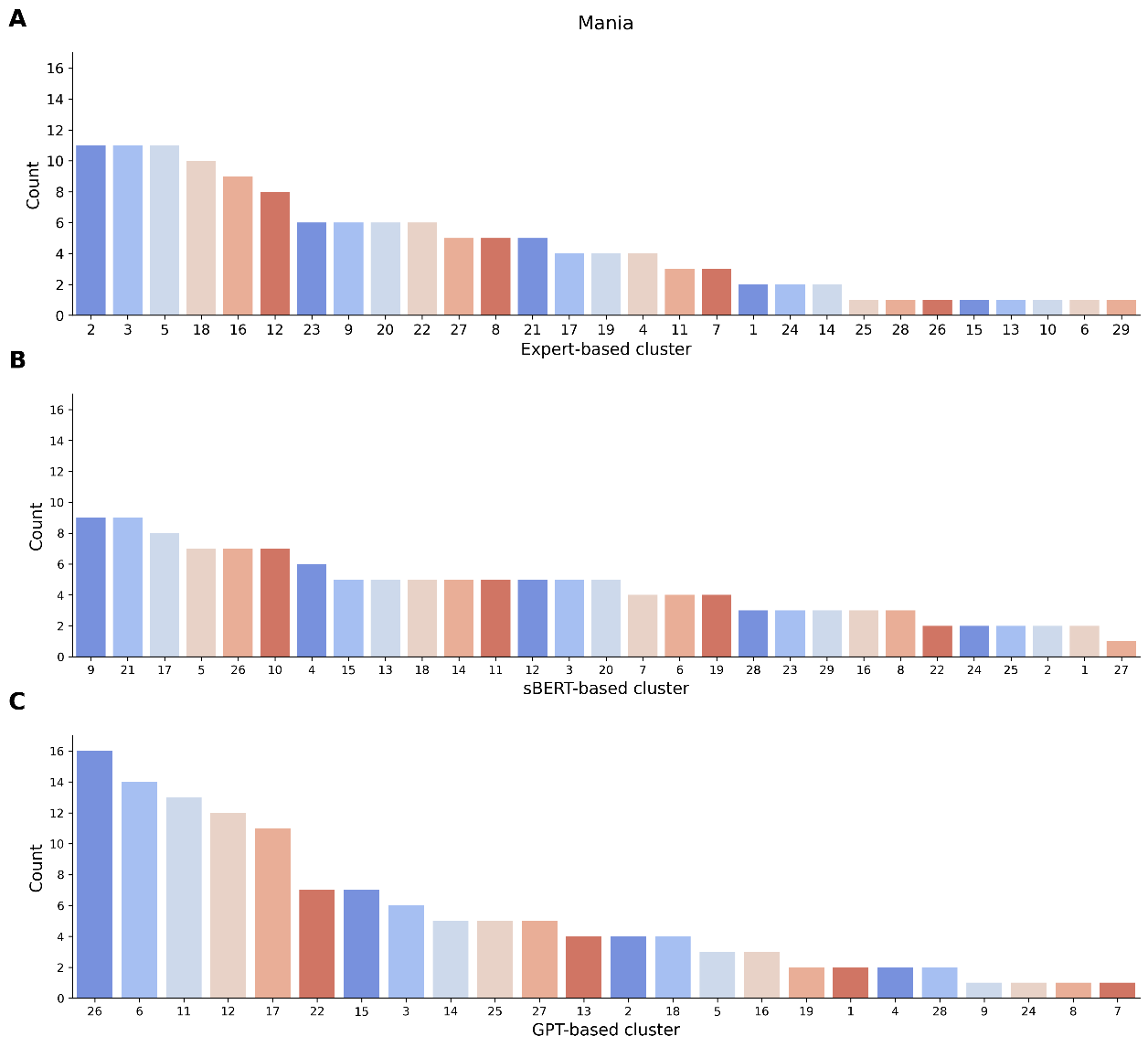


**Figure S4.** Number of items of mania questionnaires assigned to each cluster by (**A**) the expert-based and (**B**) the embedding-based clustering methods. sBERT: Sentence - Bidirectional Encoder Representations from Transformers. GPT: Generative Pre-Trained Transformer.


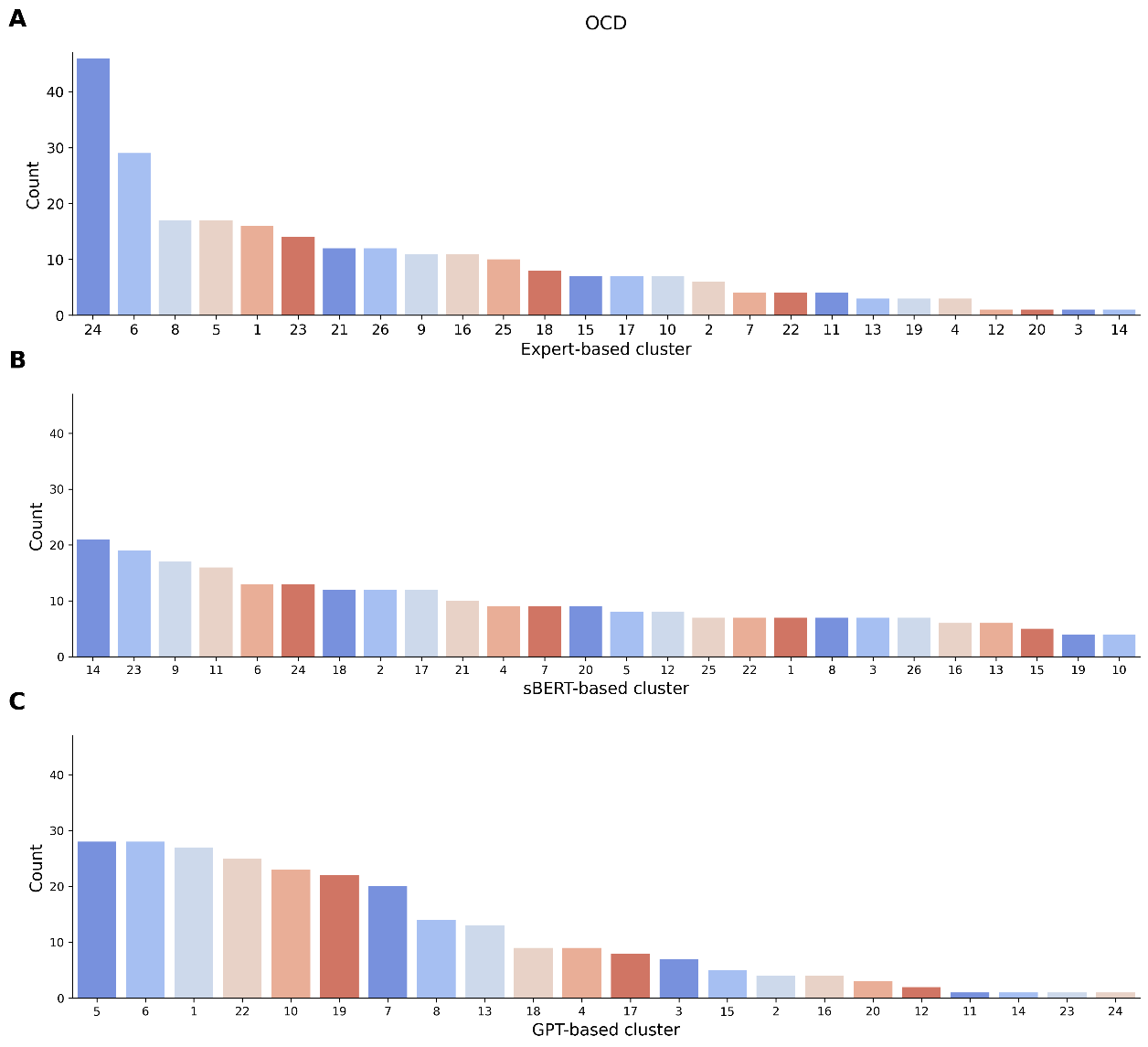


**Figure S5.** Number of items of obsessive-compulsive disorder (OCD) questionnaires assigned to each cluster by (**A**) the expert-based and (**B**) the embedding-based clustering methods. sBERT: Sentence - Bidirectional Encoder Representations from Transformers. GPT: Generative Pre-Trained Transformer.


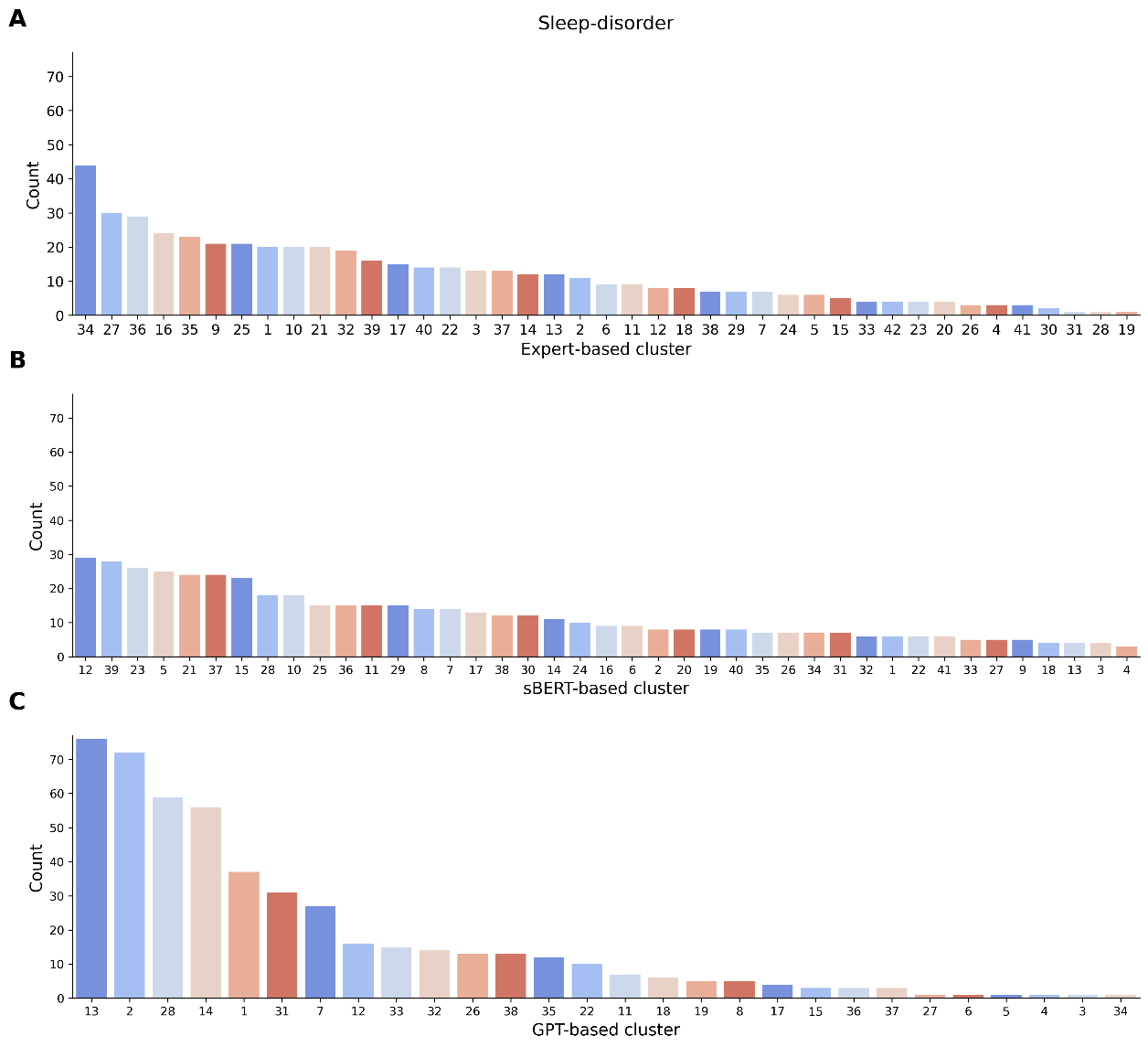


**Figure S6.** Number of items of sleep-disorder questionnaires assigned to each cluster by (**A**) the expert-based and (**B**) the embedding-based clustering methods. sBERT: Sentence - Bidirectional Encoder Representations from Transformers. GPT: Generative Pre-Trained Transformer.

Similarity between expert clusterings, expert-based and sBERT-based clusterings, and expert-based and GPT-based clusterings indicated by the adjusted rand index (ARI) is illustrated in Figure S7.


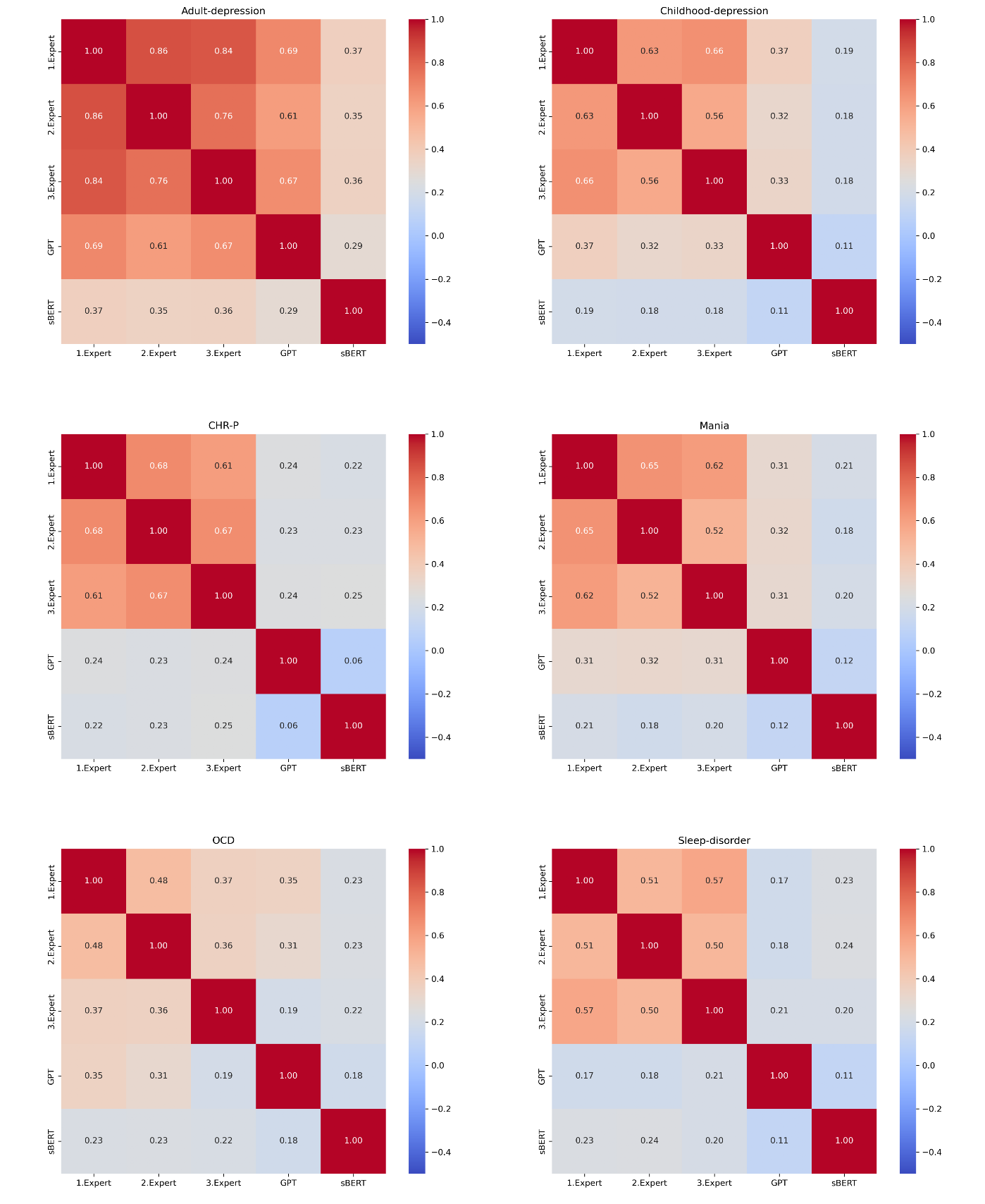


**Figure S7.** Adjusted rand index comparing clustering solutions of questionnaire items between expert raters, between expert-based to sBERT-based clustering solutions, and between expert-based to GPT-based clustering solutions. CHR-P: clinical high risk for psychosis. OCD: obsessive-compulsive disorder. sBERT: Sentence - Bidirectional Encoder Representations from Transformers. GPT: Generative Pre-Trained Transformer.
